# Changes over time in the health and functioning of older people moving into care homes: analysis of data from the English Longitudinal Study of Ageing

**DOI:** 10.1093/ageing/afx158

**Published:** 2018-03-01

**Authors:** Isabel Green, Daniel Stow, Fiona E. Matthews, Barbara Hanratty

**Affiliations:** 1 Institute of Health and Society, Newcastle University, Newcastle upon Tyne, UK; 2 MRC Biostatistics Unit, University of Cambridge, Cambridge UK

**Keywords:** ageing, nursing homes, care homes, functional impairment, older people

## Abstract

**Background:**

the number of people requiring care home support is projected to rise in future years, but little information is available on the needs of new care home residents.

**Objective:**

to measure the health and functioning of people moving into care homes and how they have changed between 2002 and 2015.

**Setting:**

English Longitudinal Study of Ageing.

**Participants:**

two hundred fifty-four of the 313 (1.99%) individuals who moved from the community into a care home, and were interviewed in the survey wave prior to entry.

**Main outcome measures:**

changes over time for number of health conditions and functional deficits (deficits in activities of daily living (ADL), and instrumental ADLs (IADLs)), assessed in the survey wave prior to admission.

**Results:**

over time there were significant increases in the total number of health conditions and functional deficits amongst soon to be care home entrants (*P* = 0.0011), the proportion with high blood pressure (OR 1.37, 95% CI: 1.17–1.62, *P* < 0.0001), memory problems (OR 1.33, 95% CI: 1.11–1.61, *P* = 0.0021) or total number of IADL deficits (*P* = 0.008). Non-significant increases were observed in the proportion of care home entrants with cancer (OR 1.23, 95% CI: 0.93–1.65, *P* = 0.15), lung disease (OR 1.21, 95% CI: 0.85–1.75), heart disease (OR 1.12, 95% CI: 0.95–1.30) and arthritis (OR 1.11, 95% CI: 0.95–1.30). Stroke and ADL deficits did not increase. No differential ageing effect was observed.

**Conclusions:**

the support needs of care home entrants in England appear to be increasing over time. This has important implications for the provision and funding of care home places and community services.

## Introduction

Approximately 400,000 people aged over 65 years live in care homes in England [1]. As life expectancy continues to rise, the number of people in England who may need the level of support provided by a care home is projected to rise to almost 500,000 by 2030 [2]. However, the number of care home beds is not expected to rise to meet this demand and the future funding and stability of long-term care sector is uncertain [3]. Understanding the changing nature of demands on care homes and community services is important for service planning and the development of a skilled workforce.

Data from the national census show that the care home population aged between 2001 and 2011 [1]. It is difficult to determine what impact this has had on the health and functioning of residents, because there is no routine mechanism for recording the health outcomes of care home residents in England [4]. The limited available evidence suggests that residents are increasingly dependent [5], living with a growing number of long-term physical health conditions and are frequent users of NHS services [6].

The aim of this study is to describe how the health, functioning and support needs of people moving into care homes in England changed between 2002 and 2015, using data from a nationally representative study.

## Method

### Study design, setting and participants

Data were obtained from waves 1 (2002) to 7 (2015) of the English Longitudinal Study of Ageing (ELSA). The ELSA cohort consists of 15,783 individuals who are representative of the English population aged 50 and over. Participants are interviewed every 2 years. ELSA does not recruit new members from care homes, but does continue to attempt to collect data from existing participants who move into care homes. We identified individuals who were recorded as living in a care home and extracted health and sociodemographic information from the preceding interview wave. Individuals were excluded if no survey data were available from the wave prior to being recorded as living in a care home.

### Study variables

The ELSA harmonised data set contains over 2,000 variables derived from ELSA interviews. Full details of the study and construction of variables is published elsewhere [7]. To examine memory problems, we used a variable corresponding to respondents’ answers to having ever been diagnosed with a memory-related disease or dementia. Individuals who rated their health as fair, poor or very poor (as opposed to excellent, very good or good) were categorised as having poor self-rated health. We also investigated self-reported arthritis, diabetes, cancer, lung disease, stroke and heart disease. To measure functional impairment, we classified individuals into three levels: those with no difficulties with any activities of daily living (ADL) or instrumental ADL (IADL) were classified as no functional impairment; individuals with no ADL difficulties, but with one or more IADL difficulty as having mild functional impairment and individuals with one or more ADL difficulty as having moderate functional impairment. The total burden of health conditions and functional impairment was calculated as the sum of these variables. (Physical and cognitive health deficits were coded as 0/1, the functional items were coded 0 for no functional impairment, 1 for mild and 2 for moderate impairment). In addition, the number of IADL or ADL impairments were also investigated separately (total out of 5).

### Analysis

Linear regression was used to model the relationship between total number of health conditions and functional impairments and wave of entry to care homes. Supplemental analyses were carried out to examine changes over time for each health condition and measure of functional deficit separately. Binary measures were examined using logistic regression, with linear regression for continuous measures. Study wave was entered as a continuous variable into all models to reflect time. The ELSA cohort was refreshed with the addition of younger individuals at waves 3, 4 and 6 to address selection bias due to attrition, and to ensure adequate representation of people age 50–52 years. To demonstrate that any trends observed in our analysis were not an artefact of the ageing cohort, all models were adjusted for age. To investigate whether the care home population has aged differentially, two-way analysis of variance was carried out on age at baseline (the age at which they were 1st seen by an ELSA interviewer), sex, and wave prior to entry to a care home, with an interaction between sex and wave. All analyses were conducted using R software version 3.3.0. *P* values ≤ 0.05 were taken to indicate conventional significance.

## Results

A total of 313 (1.99%) individuals moved from the community to a care home across all waves of data collection (2002–2015). Of these individuals, 254 had interview data available from the wave prior to being recorded as living in a care home. Twenty-three percent of interviews were conducted via proxies. Table 1 lists characteristics of the individuals eligible for inclusion in our analysis.

A majority of individuals entering care homes were female, and this did not change over time. Female care home entrants were older than men across all waves (*P* < 0.0001) and no differential ageing was observed (p_interaction_ = 0.66).

The total number of health conditions and functional deficits in soon to be care home entrants has increased with time (0.12 items per year, 95% CI: 0.05–0.19, *P* = 0.0011). Breaking this down into individual measures of health and function showed that the proportion of ELSA soon to be care home entrants with a memory problem increased significantly over time (OR 1.29, 95% CI: 1.14–1.55, *P* = 0.0052) as with high blood pressure (OR 1.37, 95% CI: 1.17–1.62, *P* < 0.0001) and self-reported IADLs (0.09 (95% CI: 0.02–0.16) more IADL deficits per year, *P* = 0.0080). Other binary variables showed similar (though non-significant) increases over time in the proportion of soon to be care home entrants with cancer (OR 1.29, 95% CI: 0.99–1.72, *P* = 0.0649), lung disease (OR 1.18, 95% CI: 0.84–1.70, *P* = 0.35), heart disease (OR 1.14, 95% CI: 0.98–1.33, *P* = 0.087) and arthritis (OR 1.13, 95% CI: 0.97–1.31, *P* = 0.11). There was no clear increase over time in the prevalence of stroke (OR 1.02, 95% CI: 0.86–1.22, *P* = 0.79), having poor self-rated health (OR 0.99, 95% CI: 0.83–1.18, *P* = 0.93) or in the number of ADL deficits (*P* = 0.63). For further details of the models described, see Supplementary data, Tables S1–S4, available at *Age and Ageing* online.

## Discussion

Using data from a nationally representative study, we have shown that the health and functioning of older adults soon to enter care homes declined between 2002 and 2015. Furthermore, this trend is not an artefact of the ageing care home population.

People soon to be admitted to care homes between 2002 and 2015 showed an increase in the total number of health conditions and functional deficits, and were increasingly likely to report memory-related diagnoses, arthritis and heart disease. Recent research has found that the number of people developing dementia in any given year has remained stable [8], and this implies that selection into the care home population is changing. Dementia and cognitive impairment are known to be associated with entry to a care home [9, 10]. Our study found that long-term physical conditions are increasingly common amongst new residents, which may reflect greater awareness or changes in diagnostic practice in primary care. Whilst long-term conditions may not be strong independent predictors of admission, they will influence need for support [9]. The prevalence of multi-morbidity in the ageing population is predicted to rise in future years. How this will affect the needs of care home residents requires ongoing monitoring.

Presentation of nationally representative data on the health and functioning of people prior to care home admission is novel, and a strength of this study. ELSA is one of the few large studies that attempts to collect data on participants after they move into care homes. However, censoring of age at 90 years in ELSA limits our understanding of the needs of the oldest old. A number of care home residents were excluded from our study because they were not interviewed in the year prior to admission. We also have no information on people who moved into a care home and died before being interviewed. Non-participation due to declining physical or mental health or death is likely to underestimate the prevalence of poor health and functioning in our analysis.

## Implications

An understanding of how the health and functioning of care home residents have changed over time is essential to allow policy makers to anticipate future needs. If the size of the care home population is stable but their needs are increasing, this implies that more people with high level needs are continuing to live in their own homes. Ensuring that both care homes and community services are equipped to meet the consequences of an ageing population should be a priority.

## Supplementary Material

Supplementary data are available at *Age and Ageing* online.

Supplementary information

## Figures and Tables

**Table 1:** Characteristics of ELSA participants who moved into a care home by the next wave of data collection, by wave.

	Wave											
	1		2		3		4		5		6	
Number of care home entrants with data	54		37		48		43		39		33	
						
Age, years: median (range)	81.0 (58–91)		83.0 (65–91)		83.5 (53–91)		84.0 (63–91)		85.0 (67–91)		85.0 (67–91)	
	*n*	%	*n*	%	*n*	%	*n*	%	*n*	%	*n*	%
Female	36	66.7	25	67.6	34	70.8	28	65.1	32	82.1	19	57.6
Living alone	29	53.7	22	59.5	27	56.3	24	55.8	22	56.4	20	60.6
Unmarried	41	75.9	28	75.7	29	60.4	25	58.1	28	71.8	20	60.6
Poor self-rated health	29	53.7	20	54.1	16	33.3	14	32.6	16	41.0	13	39.4
Mild disability	7	13.0	11	29.7	4	8.3	5	11.6	10	25.6	4	12.1
Moderate disability	27	50.0	17	45.9	31	64.6	28	65.1	22	56.4	17	51.5
Memory problems	6	11.1	7	18.9	11	22.9	13	30.2	12	30.8	10	30.3
Arthritis	21	38.9	17	45.9	23	47.9	23	53.5	19	48.7	20	60.6
Heart disease	18	33.3	14	37.8	12	25.0	18	41.9	19	48.7	16	48.5
Lung disease	2	3.7	1	2.7	2	4.2	3	7.0	1	2.6	3	9.1
Cancer	5	9.3	0	0.0	2	4.2	3	7.0	7	17.9	5	15.2
Stroke	9	16.7	10	27.0	11	22.9	18	41.9	4	10.3	7	21.2
High blood pressure	23	42.6	15	40.5	26	54.2	29	67.4	23	59.0	25	75.8
Number IADL items (mean, SD)	1.28	(1.54)	1.84	(1.49)	1.85	(1.57)	2.33	(1.71)	2.10	(1.56)	2.21	(1.67)
Number ADL items (mean, SD)	1.36	(1.54)	1.05	(1.49)	1.54	(1.57)	1.72	(1.71)	1.31	(1.56)	1.36	(1.67)
Total item score (mean, SD)	3.37	(2.00)	3.57	(1.66)	3.60	(1.84)	4.47	(1.87)	4.15	(1.81)	4.45	(2.22)

## Key points

- The care home population is stable in size, but it is ageing.
- There are no up to date or nationally representative analyses of the health and functioning of care home entrants over time.
- The health and functioning of care home entrants declined between 2002 and 2015. Observed changes were not an artefact of an ageing care home population.
- Care homes need to be equipped to meet the increasing support needs of their residents.
